# Joint genomic evaluation of French dairy cattle breeds using multiple-trait models

**DOI:** 10.1186/1297-9686-44-39

**Published:** 2012-12-07

**Authors:** Sofiene Karoui, María Jesús Carabaño, Clara Díaz, Andrés Legarra

**Affiliations:** 1 INIA, Depto. de Mejora Genética Animal, Ctra. de La Coruña Km 7.5, Madrid, 28040, Spain; 2 INRA, UR 631 SAGA, Castanet Tolosan, F-31326, France

## Abstract

**Background:**

Using a multi-breed reference population might be a way of increasing the accuracy of genomic breeding values in small breeds. Models involving mixed-breed data do not take into account the fact that marker effects may differ among breeds. This study was aimed at investigating the impact on accuracy of increasing the number of genotyped candidates in the training set by using a multi-breed reference population, in contrast to single-breed genomic evaluations.

**Methods:**

Three traits (milk production, fat content and female fertility) were analyzed by genomic mixed linear models and Bayesian methodology. Three breeds of French dairy cattle were used: Holstein, Montbéliarde and Normande with 2976, 950 and 970 bulls in the training population, respectively and 964, 222 and 248 bulls in the validation population, respectively. All animals were genotyped with the Illumina Bovine SNP50 array. Accuracy of genomic breeding values was evaluated under three scenarios for the correlation of genomic breeding values between breeds (r_g_): uncorrelated (1), r_g_ = 0; estimated r_g_ (2); high, r_g_ = 0.95 (3). Accuracy and bias of predictions obtained in the validation population with the multi-breed training set were assessed by the coefficient of determination (R^2^) and by the regression coefficient of daughter yield deviations of validation bulls on their predicted genomic breeding values, respectively.

**Results:**

The genetic variation captured by the markers for each trait was similar to that estimated for routine pedigree-based genetic evaluation. Posterior means for r_g_ ranged from −0.01 for fertility between Montbéliarde and Normande to 0.79 for milk yield between Montbéliarde and Holstein. Differences in R^2^ between the three scenarios were notable only for fat content in the Montbéliarde breed: from 0.27 in scenario (1) to 0.33 in scenarios (2) and (3). Accuracies for fertility were lower than for other traits.

**Conclusions:**

Using a multi-breed reference population resulted in small or no increases in accuracy. Only the breed with a small data set and large genetic correlation with the breed with a large data set showed increased accuracy for the traits with moderate (milk) to high (fat content) heritability. No benefit was observed for fertility, a lowly heritable trait.

## Background

Increasing the accuracy of the prediction of breeding values has become a major objective in genomic selection (GS). The success of GS depends on many factors [1,2], some of which cannot be easily controlled, such as linkage disequilibrium (LD) between markers and quantitative trait loci (QTL), the size of the training dataset, and marker densities at a given cost. The heritability of the trait is also a limiting factor.

It has been observed that accuracy increases with increasing size of the training data [3,4]. For this reason, joint genomic evaluations based on data from a consortium of countries are being carried out for a given breed, such as for the Holstein breed in the EuroGenomics [3] and North-American consortiums [1] and for the Brown Swiss breed in the Intergenomics consortium [5]. However, for local breeds and/or of small size, an alternative is to train on data from several breeds simultaneously [6,7]. A multi-breed reference population could be an appealing solution to increase the reference population size, especially if some of the analyzed breeds have small population sizes. However, most multi-breed studies assume that marker effects are the same across populations [6,8-10]. This assumption, albeit useful, is hardly tenable, because it assumes that the pattern of linkage disequilibrium is the same in each breed. Also, the underlying architecture (QTL frequencies and interactions) does not need to be the same between breeds. Furthermore, if breeds are not crossed (which is the case for the above studies and in this one), there is no interest in estimating breeding values of composite animals on a hypothetical “multiple breed” base population. Quite the opposite, dairy cattle breeders are interested in estimated breeding values (EBV) expressed on the scale of each pure breed. Several recent studies have used approaches that overcome the assumption of equal marker effects across populations. Makgahlela et al. [11] proposed to define multiple breeds as an admixture of populations by taking breed proportions into account in the context of a random regression model. However, in most cases, this admixture of breeds does not exist or cannot be identified. Varona et al. [12] used models that allow for SNP (single nucleotide polymorphisms) effects to differ in variance, value and sign between populations in heteroscedastic or multiple trait settings. In this work, we investigated the impact on accuracy of increasing the size of the training set by using a multi-breed French reference population under differing assumptions for the genetic correlations between breeds, in contrast to single-breed genomic evaluation.

Three traits (milk yield, fat content and female fertility defined as non return rate at 56 days), which have different genetic backgrounds were analyzed in three major French dairy cattle breeds: Montbéliarde (M), Normande (N) and Holstein (H).

## Methods

### Estimation of genetic correlation between breeds using genomic information

Varona et al. [12] suggested that the SNP effects could be modeled assuming that there is a genetic correlation of SNP effects across breeds. These authors modeled breeding values (**u**) as a sum of marker effects (**g**) so that **u=Zg** and ordered by breed (breeds 1 and 2 for illustration):

$$u=\{\begin{array}{c} u_{breed1}\\ u_{breed2} \end{array}=\{\begin{array}{c} Z_{breed1}g_{breed1}\\ Z_{breed2}g_{breed2} \end{array}=Zg$$

Marker effects were assumed to have a multivariate distribution:

$$\begin{array}{c} \left(\begin{array}{c} g_{\text{breed}1}\\ g_{\text{breed}2} \end{array}\right)∼N\;\left[\left(\begin{array}{c} 0\\ 0 \end{array}\right),\left(\begin{array}{cc} I\sigma_{g1}^{2} & I\sigma_{g1,2}\\ I\sigma_{g2,1} & I\sigma_{g2}^{2} \end{array}\right)\right]\\ \;=N\left(0,B⊗I\right) \end{array}$$

Where **I** is an identity matrix of order equal to the number of SNP markers and **B** is a 2 x 2 breed covariance matrix for SNP effects.

VanRaden [13] (and also [14]) showed how models that assume normality of marker effects (the so-called “BLUP-SNP”, [15]) can be transformed into equivalent BLUP animal models (usually known as **G**BLUP) that use a “genomic” relationship matrix, usually termed **G**, rather than a pedigree-based relationship matrix. Matrix **G** is an estimator of the “true” proportions of genes that are identical by descent between individuals [16,17]. Based on this equivalence, the model by Varona et al. [12] can be transformed into the following model:

$$\begin{array}{c} u=\left\{\begin{array}{c} u_{breed1}\\ u_{breed2} \end{array}∼N\;\left[\left(\begin{array}{c} 0\\ 0 \end{array}\right),\left(\begin{array}{cc} G\sigma_{u1}^{2} & G\sigma_{u1,2}\\ G\sigma_{u2,1} & G\sigma_{u2}^{2} \end{array}\right)\right]\right)\\ \;=N\left(0,G_{0}⊗G\right)\text{,} \end{array}$$

Where **u**_breedi_ is a vector of genomic breeding values (GBV) for breed i, **G** is a matrix of genomic relationships (animals in all breeds), and **G**_0_ is a matrix of variances and covariances associated to GBV in each breed for a given trait. This model is, thus, a multiple-trait model with two “pseudo-traits”, reflecting the breeding value for the trait in breeds 1 and 2. This model resembles the MACE model [18] in which the breeding values of each bull in different countries are seen as different, correlated traits. In this model, the genetic distance (for each trait) between breeds is quantified by the genetic correlations between **u**_breed1_ and **u**_breed2_ (similar to the genetic correlations across countries in MACE). Note that if *σ*_*u*1_^2^ = *σ*_*u*2_^2^ = *σ*_*u*1,2_, the model reduces to the regular GBLUP model as used, for instance, by Hayes et al. [6] or [9]. In addition, if *σ*_*u*1,2_ = 0, the model reduces to two independent GBLUP models, one for each breed. In addition to the theoretical appeal, one advantage of a multi-trait GBLUP model is the possibility of using standard estimators and existing software to predict breeding values and estimate variance components.

### Data

Table 1 gives details on the constitution of the different validation and reference populations. The reference population included data on 4896 bulls from the M, N and H breeds and was used to estimate genetic parameters and GBV. Thus, the multi-breed reference population included M (n = 950), N (n = 970) and H (n = 2976) bulls with a large number of daughters. The average equivalent daughter contributions (EDC) ranged from 407 to 513 for M and H, respectively. The validation populations included the youngest bulls (born after year 2004) from each breed that had at least 40 daughters in production since October 2009. These bulls were used to evaluate the accuracy of the genomic estimated breeding values (GEBV).

**Table 1:** Number of animals genotyped by breed and size of the training and validation datasets

**Dataset**	**Montbéliarde**	**Normande**	**Holstein**	**Total number**
Training	950	970	2976	4896
Validation	222	248	964	1434
Total	1172	1218	3940	6330

All bulls were genotyped with the 50k SNP using the Illumina Bovine array. The SNP were filtered by extreme Hardy-Weinberg disequilibrium (p < 10^-6^) and Mendelian inconsistencies (the genotype of the father was deleted if more than 20% of his progeny showed contradiction). Editing was within-breed. Genotypes of the three breeds were merged, including only SNP which segregated (minor allele frequency > 3%) in each breed. In the final data set, only those loci fulfilling all requirements in all breeds were considered. Finally, 43 852 SNP were used. Pseudo-phenotypes for each bull were daughter yield deviations (DYD), as in VanRaden and Wiggans [19], with weights corresponding to the equivalent daughter contributions for each bull.

### Models

In all analyses, a given trait (i.e., milk production) was considered a different trait for each breed. To avoid confusion, these will be referred to as traits (milk production, fat content, fertility) and as scales (breeding values on the M, N or H scale).

In the first analysis, we estimated genetic variances and correlations between breeds and the heritability of each trait and each breed using a combined data set including the three breeds. For computational reasons (see later), instead of the multiple-trait model (MTM), an almost equivalent Random Regression Model (RRM) was used, similar to that used by [11]. The general equation for this model was:

$$y=Xb+W_{M}u_{M}+W_{N}u_{N}+W_{\text{H}}u_{\text{H}}+\varepsilon$$

Where, **y** is a vector of 2*DYD; **X** is a matrix that allocates each DYD to a breed and **b** a vector of average breed effects; **W**_i_ are design matrices allocating DYD to GBV (**u**_M_, **u**_N_ and **u**_H_) for the M, N and H scales consecutively. For example, the equation corresponding to bull *t* of breed H, will have a value of 1 in the (*t*,*t*) position of **W**_H_ and 0 in **W**_M_ and **W**_N_, since no bull has daughters in several breeds. Vector **ε** is a vector of uncorrelated random normal pseudo-errors (“pseudo”, because they include Mendelian sampling effects of the daughters and part of the breeding value of the mates). Homogeneous pseudo-error variances were assumed across breeds. The co(variance) structure for GBV, **u**_i,i=M,N,H_, for one trait was:

$$\begin{array}{c} \text{Var}\;\left(\begin{array}{c} u_{M}\\ u_{N}\\ u_{\text{H}} \end{array}\right)=G_{0}⊗\text{G;}\\ \;G_{0}=\left(\begin{array}{ccc} {\text{σ}}_{\text{uM}}^{2} & {\text{σ}}_{\text{uM,N}} & {\text{σ}}_{\text{uM,H}}\\ {\text{σ}}_{\text{uN,M}} & {\text{σ}}_{\text{uN}}^{2} & {\text{σ}}_{\text{uN,H}}\\ {\text{σ}}_{\text{uH,M}} & {\text{σ}}_{\text{uH,N}} & {\text{σ}}_{\text{uH}}^{2} \end{array}\right)\;\text{;}\\ \;G=\;\left(\begin{array}{ccc} G_{M} & G_{\text{M,N}} & G_{\text{M,H}}\\ G_{\text{N,M}} & G_{N} & G_{\text{N,H}}\\ G_{\text{H,M}} & G_{\text{H,N}} & G_{\text{H}} \end{array}\right) \end{array}$$

Where, **G**_**0**_ is the matrix of (co) variances of GBV in each of the three scales, M,N,H, for a given trait, named as genetic (co)variances henceforth; **G** is the genomic relationship matrix relating animals of the same and different breeds. The correlation of GBV in different scales for a given trait is denoted by r_g_, and it will be named as genetic correlation between breeds henceforth.

Matrix **G** was created as in VanRaden [13]:

$$G=0.95^{*}\frac{zz'}{2\sum_{i^{{\text{p}}_{i}{\text{q}}_{i}}}}+0.05I\text{,}$$

Where **Z** is a centered incidence matrix of genotype covariates (0/1/2); 2 ∑ p_i_ q_i_ is a scaling parameter in which p_i_ and q_i_ are the allelic frequencies for SNP i (i = 1: 43852), which were computed across breeds; **I** is an identity matrix (included in order to make **G** invertible). Matrix **I** could have been replaced by **A**, following VanRaden et al. [20], but this is not expected to affect results considering the low weight assigned to **I**.

To implement this model, the regular relationship matrix was replaced by **G** using facilities in the Blupf90 series of programs [21,22]. Variance and covariance components in the RRM were estimated using Bayesian procedures via Gibbs sampling by the Gibbs2f90 program [23]. Moreover, estimates of genetic correlations between breeds were computed from the corresponding estimates of the genetic (co)variance components. The interest in using the RRM with Gibbs sampling rather than, e.g., REML or a multiple-trait model, was the fact that, on one hand, the relationship matrix needed to be stored just once (in contrast to regular REML, for instance), and on the other hand, no “data augmentation” of missing traits was needed with the RRM, in contrast to using regular Gibbs sampling with a multiple-trait model. Both of these resulted in large reductions in computing time and memory requirements. For instance, storing **G** (which is a 6330 x 6330 dense matrix) for the MTM would take nine times as much space.

The Gibbs sampler was run for a total of 20 000 iterations. The first 4000 iterations were discarded as burn-in. Convergence was checked visually and by the Geweke diagnostic of the Markov chain [24]. Posterior means of genetic variances for each trait and for each breed and of the correlation between breeds were computed. After the parameters were estimated, the (co)variances in the model were fixed at their estimates and the RRM was used in a GBLUP analysis to estimate the GEBV of all genotyped candidates in the validation dataset.

In a second set of analyses, BLUP with a multi-breed genomic relationship matrix (GBLUP) was applied to estimate the GBV of all genotyped bulls using the following MTM:

$$y_{i}=b_{i}+W_{i}u_{i}+\varepsilon_{i}\text{,}$$

Where **y**_i_ is a vector of 2*DYD for breed i = {M,N,H}. In this model, each record is allocated to its breed-specific effects and breeding values.

The covariance structure of **u** was as for the RRM and estimated (co)variances obtained with the RRM were used in the MTM to estimate the corresponding GEBV. However, in the MTM, different residual variances for each breed were used:

$$\text{Var}\;\left(\begin{array}{c} \varepsilon_{M}\\ \varepsilon_{N}\\ \varepsilon_{\text{H}} \end{array}\right)=R_{\text{o}}⊗I;\;R_{\text{o}}=\left(\begin{array}{ccc} \sigma_{\mathrm{\varepsilon M}}^{2} & 0 & 0\\ 0 & \sigma_{\mathrm{\varepsilon N}}^{2} & 0\\ 0 & 0 & \sigma_{\mathrm{\varepsilon H}}^{2} \end{array}\right)$$

Because 2*DYD is pre-corrected data, its pseudo-residual variance is not the same as the actual residual variance. Thus, we used *σ*_*ɛ*,*i*_^2^ = (4*σ*_*ɛ*,i_^*2^ + 2*σ*_*u*,i_^*2^), where the σ^*^ indicates values from routine genetic evaluations for these breeds (S. Fritz, UNCEIA, Jouy-en-Josas, personal communication).

In both models (RRM or MTM), EDC were used as weighting factors and the GEBV were computed using BLUP90iod2 modified by Aguilar et al. [21].

### Accuracy of GEBV

For each model (MTM and RRM), three scenarios for the genetic correlation between breeds, r_g_, were assumed to compare the accuracy of GEBV. In scenario 1, r_g_ was set to zero to simulate a situation where breeds were uncorrelated, which is equivalent to performing single-breed evaluations. In scenario 2, the estimated value for r_g_ was used and in scenario 3, r_g_ was set to 0.95, which is equivalent to the assumption that the population is close to homogenous (r_g_ = 1) [6,9].

Accuracy and bias of the GEBV were assessed in the validation datasets, separately for each breed, by the coefficient of determination (R^2^) and the estimated linear regression coefficients, *δ*_0_ (intercept) and *δ*_1_ (linear term) of the linear regression of 2*DYD on GEBV, weighted by the corresponding equivalent number of daughters (EDC), respectively.

## Results

### Distribution of genomic relationship coefficients within and between breeds

**Figure 1:**
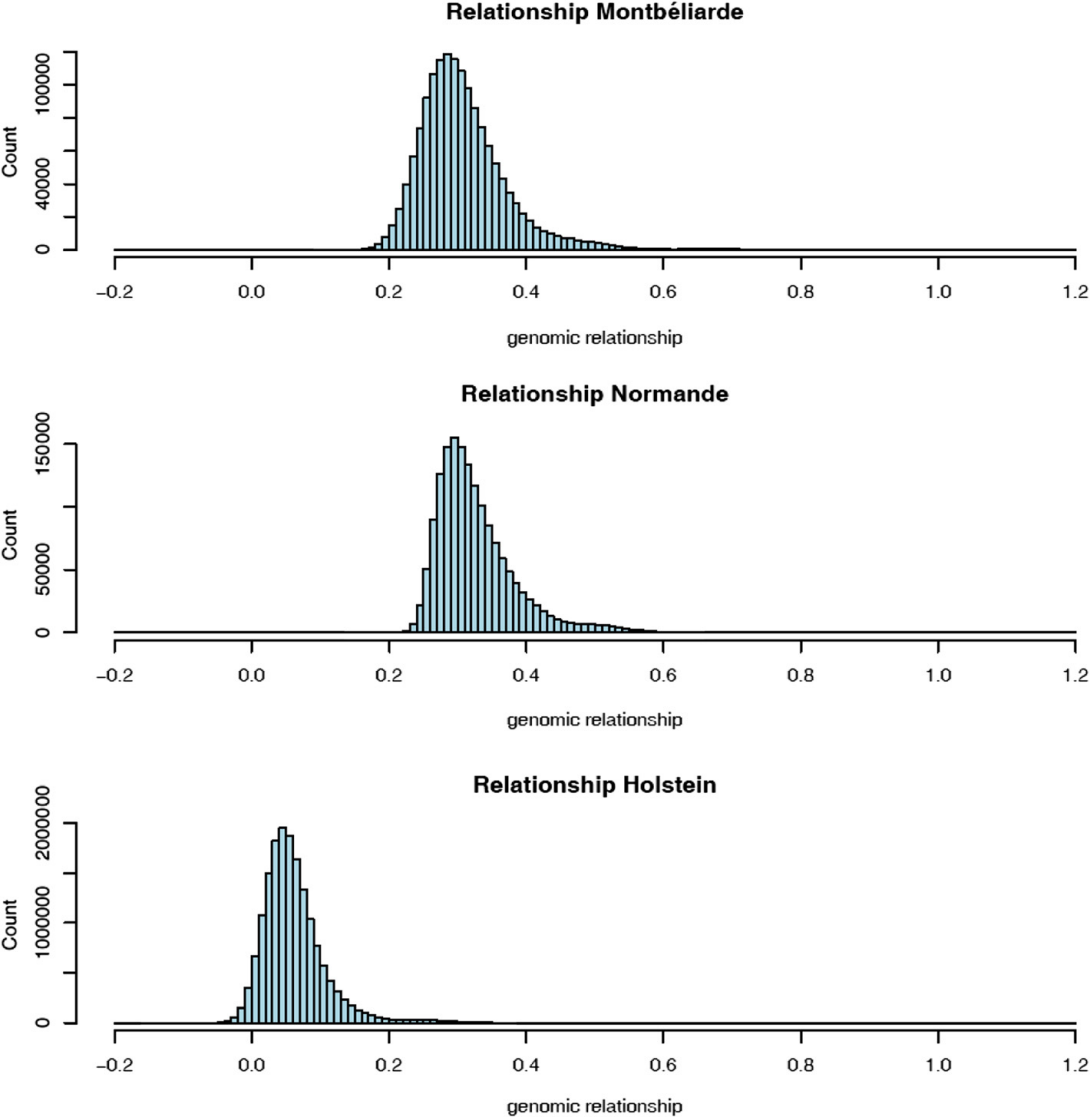
Distribution of genomic relationship coefficients within breeds.

**Figure 2:**
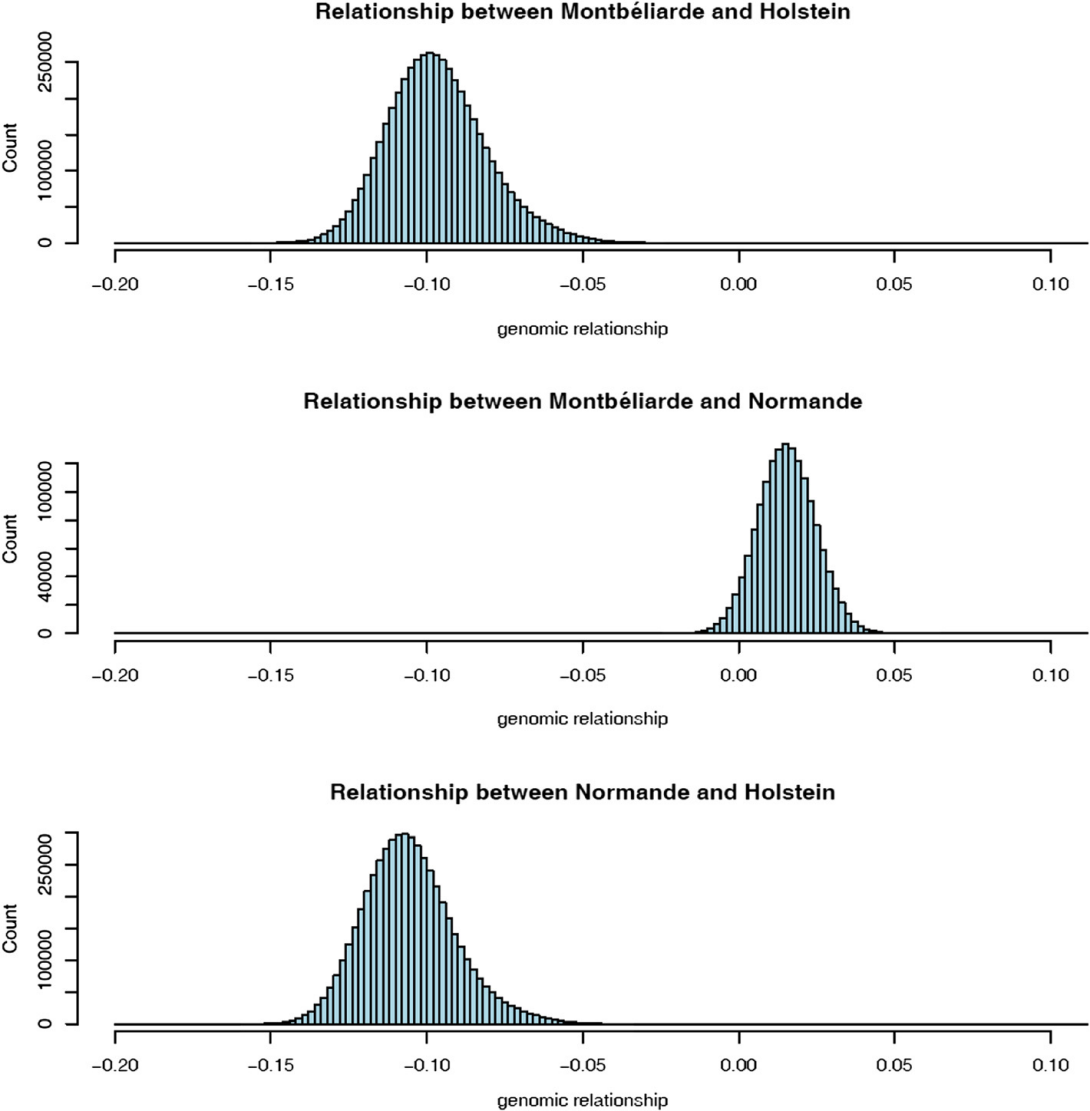
Distribution of genomic relationship coefficients between breeds.

Figures 1 and 2 show the distributions of genomic relationship coefficients within and between breeds, respectively. Figure 1 shows a higher level of relationship within the M and N breeds compared with breed H. This might be due to the larger number of individuals in breed H than in the N and M breeds, because allele frequencies were computed considering all animals. Using breed-specific allele frequencies is expected to give different results (e.g., [20]). Pedigree relationships were ascertained as well, resulting in an average within-breed relationship of 0.10. The choice of allele frequencies to be used may depend upon the goals of the analyses [20] but the effect of this choice on the results of genomic evaluation is still an open issue, particularly in the multi-breed context. Figure 2 shows a moderate level of genomic relationships between breeds compared to the within-breed relationships, as expected.

### Variance components and heritability estimates

Table 2 contains estimates of genetic variances (by breed) and pseudo-error variances for milk production, fat content and female fertility estimated in the multi-breed reference population using RRM. Genetic variance estimates were similar to those used in the routine genetic evaluation (S Fritz, UNCEIA, Jouy-en-Josas, personal communication) and the latter were included in the 95% high probability density regions (HPD95%) interval of the estimates from the genomic data, except for the genetic variance for fertility in breed H. Estimated posterior genetic variances showed a narrow HPD95% interval, indicating a high precision of the estimates using the molecular information. Pseudo-error variances differed from the residual variances used in routine genetic evaluation (not shown in the table). This result is explained by the use of the 2*DYD as a pseudo-phenotype, hence the use of the term pseudo-error variance.

**Table 2:** **Posterior means **(*σ*^2^) **and HPD95% intervals for each breed and pseudo-error variances** (*σ*^2^*ε*) **estimated for three traits**

**Trait**	***σ***^**2**^**Montbéliarde**	***σ***^**2**^**Normande**	***σ***^**2**^**Holstein**	***σ***^**2**^***ε***
	**[HPD95%]**	**[HPD95%]**	**[HPD95%]**	**[HPD95%]**
Milk	0.420 / 0.423*	0.383 / 0.358*	0.532 / 0.512*	1.866
	[0.369; 0.473]	[0.336; 0.428]	[0.487; 0.575]	[1.570; 2.141]
Fat content	5.566 / 5.115*	7.950 / 7.642*	12.692 / 12.225*	14.95
	[4.876; 6.250]	[6.898; 8.770]	[11.810; 13.700]	[12.39; 17.89]
Fertility	32.775 / 34.342*	41.505 /41.145*	57.055/ 47.321*	2238.48
	[25.960; 38.660]	[33.450; 50.150]	[50.020; 63.430]	[2078; 2408]

*σ*^2^ and *σ*^2^*ε* estimated by a multi-breed reference population for milk production, fat content and female fertility using a random regression model; *variances used in routine genetic evaluations.

Posterior means and HPD95% intervals of the heritability for each trait and breed are in Table 3. Heritabilities were calculated using as genetic variances the GBV variance estimated in the RRM. The phenotypic variance was obtained subtracting the “true” residual variance from the pseudo-error variance estimate and adding the variance of the permanent environmental effect of the cows used in the routine genetic evaluation (S Fritz, UNCEIA, Jouy-en-Josas, personal communication) , which was not estimable in our data because our DYD are “free” of permanent environmental effects. Heritabilities estimated by the RRM were then rather similar compared with those used in routine genetic evaluation.

**Table 3:** Posterior means and HPD95% intervals of the heritability estimated by a multi-breed reference population

**Trait**	**Montbéliarde**	**Normande**	**Holstein**
	**[HPD95%]**	**[HPD95%]**	**[HPD95%]**
Milk	0.33 / 0.30*	0.33/ 0.30*	0.37 / 0.30*
	[0.29 ; 0.38]	[0.29 ; 0.38]	[0.33 ; 0.41]
Fat content	0.42/ 0.50*	0.44 / 0.50*	0.48 / 0.50*
	[0.36 ; 0.46]	[0.39 ; 0.49]	[0.45 ; 0.51]
Fertility	0.05 / 0.02*	0.06 / 0.02*	0.08 / 0.02*
	[0.04 ; 0.06]	[0.04 ; 0.07]	[0.06 ; 0.09]

*Heritabilities estimated in routine genetic evaluation.

### Estimation of genetic correlations between breeds

Table 4 shows the posterior means of the genetic correlations between breeds for each trait when combining information from the M, N, and H reference populations. Posterior means of genetic correlations for milk production and fat content were moderately high, particularly for correlations between breeds M and H (0.66 and 0.79 for fat content and milk production, respectively), whereas genetic correlations between breeds for female fertility were relatively low (−0.01; 0.39).

**Table 4:** Posterior means and HPD95% intervals of genetic correlations between breeds estimated by a multi-breed reference population

**Trait**	**Montbéliarde-Normande**	**Montbéliarde-Holstein**	**Normande-Holstein**
	**[HPD95%]**	**[HPD95%]**	**[HPD95%]**
Milk	0.46	0.79	0.38
	[0.26 ; 0.65]	[0.63 ; 0.93]	[0.19 ; 0.55]
Fat content	0.35	0.66	0.56
	[0.07 ; 0.64]	[0.50 ; 0.84]	[0.34 ; 0.76]
Fertility	−0.01	0.39	0.22
	[−0.50 ; 0.54]	[−0.05 ; 0.73]	[−0.15 ; 0.54]

Estimated posterior correlations showed large HPD95% intervals, especially between breed M and N and breeds N and H, whereas the genetic correlation between breeds M and H showed the narrowest HPD95% intervals. Female fertility showed the largest HPD95% intervals, indicating that the available information was not sufficient to estimate accurately the genetic correlations between breeds for this trait.

### Accuracies in prediction of the validation data set

Estimated accuracies calculated as R^2^ for the validation populations in each breed are in Table 5 for each scenario and each model (RRM vs. MTM). The R^2^ in the reference data was close to 1 for all the traits and breeds, as expected (results not shown). Estimated accuracies in the validation populations were slightly greater under the nonzero r_g_ scenarios (2 and 3), as compared to accuracies, estimated in a single-breed scenario (r_g_ = 0), for both models for milk production and fat content. The most important increase of accuracy was observed for fat content for breed M (from 0.27 with the single-breed scenario to 0.33 in the nonzero r_g_ scenarios). Female fertility was the only trait for which accuracy was not improved in any population or model when the genetic correlation between breeds was allowed to be different from zero. This result may be because of the low heritability and the smaller estimates of genetic correlations between breeds for this trait, which may indicate that fertility is biologically different between breeds.

**Table 5:** Coefficient of determination of twice of the daughter deviation yield on genomic estimated breeding values in the validation bulls

		**RRM**			**MTM**		
**Trait**	**r**_**g**_	**M**	**N**	**H**	**M**	**N**	**H**
Milk	0	0.19	0.12	0.30	0.17	0.12	0.30
	Estimated	0.21	0.13	0.31	0.19	0.13	0.30
	0.95	0.21	0.14	0.31	0.19	0.13	0.30
Fat content	0	0.27	0.39	0.51	0.27	0.39	0.47
	Estimated	0.33	0.39	0.52	0.32	0.40	0.48
	0.95	0.33	0.39	0.52	0.33	0.40	0.49
Fertility	0	0.19	0.07	0.11	0.19	0.07	0.10
	Estimated	0.19	0.07	0.11	0.20	0.07	0.10
	0.95	0.19	0.07	0.11	0.20	0.07	0.10

M: Montbéliarde; N:Normande; H: Holstein; RRM: Random Regression Model

MTM: Multiple Trait Model; r_g:_ genetic correlation between breeds.

Accuracies of GEBV were largest for the H breed for milk production (0.30 and 0.31 under RRM and MTM, respectively) and fat content (0.52 and 0.48 under RRM and MTM, respectively) traits because of the larger number of genotyped animals in this breed (Table 1). However, for fertility, the M breed had the largest accuracies (0.19 under the two models).

A small difference in accuracies was observed between the RRM and MTM models, with the RRM showing a slightly higher accuracy (Table 5).

Table 6 shows the estimated accuracies of EBV obtained from routine genetic evaluation based on pedigree for each breed (S Fritz, UNCEIA, Jouy-en-Josas, personal communication). Estimated accuracies of GEBV (Table 5) were larger than those obtained using pedigree information for milk production and fat content. For female fertility, only a small gain was observed. Again, the low heritability of this trait is the likely reason of this result.

**Table 6:** Coefficient of determination of twice of the daughter yield deviation on estimated breeding values obtained from a routine genetic evaluation

**Trait**	**Montbéliarde**	**Normande**	**Holstein**
Milk	0.08	0.09	0.14
Fat content	0.16	0.34	0.19
Fertility	0.18	0.06	0.08

The coefficient of regression of 2*DYD on GEBV (*δ*_1_) was also used to test the impact of increasing the size of the reference population using multi-breed data. The expected value of *δ*_1_ is 1, and this is desired to avoid inflation (or under-inflation) of GEBV’s of young bulls. Table 7 shows the regression coefficients estimated by the two models and for each scenario and breed. The estimates were larger for MTM than for RRM for all traits, breeds, and scenarios. Female fertility for breed M presented the worst estimate of *δ*_1_ (1.50 and 1.80 for RRM and MTM, respectively), whereas accuracies estimated by R^2^ for this breed were largest. Thus, the results show some degree of trade-off between R^2^ and *δ*_1_ used to evaluate the GEBV predictions. It is important to note that the H breed presented the best quality of predictions in terms of *δ*_1_ for all traits and breeds.

**Table 7:** Coefficient of regression of twice of the daughter deviation yield on genomic estimated breeding values of the validation bulls

		**RRM**			**MTM**		
**Trait**	**r**_**g**_	**M**	**N**	**H**	**M**	**N**	**H**
Milk	0	0.81	0.68	0.74	0.89	0.71	0.80
	Estimated	0.82	0.71	0.74	0.91	0.73	0.80
	0.95	0.81	0.69	0.73	0.91	0.74	0.80
Fat content	0	1.02	1.03	0.93	1.12	1.13	1.02
	Estimated	1.11	1.02	0.93	1.21	1.13	1.02
	0.95	1.08	0.98	0.93	1.20	1.10	1.01
Fertility	0	1.51	1.01	0.74	1.80	1.09	0.87
	Estimated	1.51	0.99	0.74	1.80	1.09	0.87
	0.95	1.48	0.91	0.73	1.78	1.05	0.86

M: Montbéliarde; N: Normande; H: Holstein; RRM: Random Regression Model

MTM: Multiple Trait Model; r_g:_ genetic correlation between breeds.

## Discussion

This study shows that the use of a multi-breed reference dairy cattle population did not have a large impact on the accuracy of prediction of GBV for young bulls. This confirms the findings of Hayes et al. [6] and also of [9,25] for multi-breed reference populations. However, using a combined H and Jersey reference population and Bayes type methods that rely on estimates of SNP effects to predict the genomic breeding values, Hayes et al. [6] found an increase of up to 17% in the accuracy of GEBV for fat yield and for fat and protein percent for young Jersey bulls. Other studies [3,4] have reported an important increase in accuracies (up to 20%) if the size of the training set increases when using one breed from different countries (international evaluation). Olson et al. [26] found a general increase of 2% from pooling U.S. and Canadian H populations and 5% for the Brown Swiss from European countries when using multiple trait methodology. Given that large or moderately large genetic correlations have been estimated for the same trait measured in different countries but on the same breed (see, e.g., [3] for Holstein populations), larger benefits in accuracy of GEBV from using a combined reference population seem to be obtained when the genetic correlations between the trait measured in different populations are larger.

In this study, a notable improvement in accuracy (6%) from using a multi-breed reference population was observed only for fat content in the M breed. The M breed showed the largest estimated genetic correlations with the H breed (0.79, 0.66 and 0.39 for milk yield, fat content and fertility, respectively). This indicates that the SNP effects are more similar between the M and H breeds than with breed N. This might be because of the introgression of Red Holstein animals in the M breed in the 1970’s (e.g. [27]). Therefore, breed M would be the one expected to obtain the largest benefits from multi-breed evaluation. Although milk yield was the trait showing the largest genetic correlation between breed M and the other breeds, the improvement in accuracy was very small (2%) for this trait. The larger response for fat content might be related to the different genetic architecture of this trait. The 50k bovine chip contains SNP that are in close LD with the DGAT1 polymorphism, which explains about 40% of the genetic variation in fat percentage in the milk of H cattle [28]. The “K” allele for DGAT1 in breed M probably originated from breed H [27] and is expected to show similar LD around it, which may explain why this trait benefits most from multi-breed evaluation; i.e., some chromosome segments of large effect that segregate in breed M are better estimated when including data on breed H. Fat content has been found to show larger benefits from the use of genomic information in other studies [6,9]. The H breed did not benefit from the large genetic correlations with the other breeds, probably because, with the larger size of the H breed reference population, the observed accuracy is close to the maximum achievable value given the existing LD. The N breed had lower estimated genetic correlations with the large H breed, and only showed minor improvements in accuracy (1-2%) from multi-breed evaluation for milk yield and fat content.

For female fertility, accuracies of GEBV in the validation populations were the same using multiple-breed or a single-breed reference population (scenarios 2 and 3 versus scenario 1), showing low sensitivity to the value of the genetic correlation. The small estimated correlations between breeds for fertility (−0.01 to 0.39) could explain the low gain in accuracies for fertility when GEBV were estimated by a multi-breed reference population. This might indicate that the LD between markers and QTL does not persist between breeds and/or that the effects of these QTL differ between breeds. In addition to no effect on accuracy, the regression coefficient of 2*DYD on GEBV was greater than one for fertility in the M breed, which indicates a severe underestimation of GEBV.

De Roos et al. [7] and (also [29]) proposed the use of a greater density of markers when the breeds that are used as a reference population are too diverged to detect enough marker-QTL relationships, such that the effect of all QTL can be captured by the SNP [30]. However, Harris et al. [31] did not find significant increase in accuracies of GEBV when a higher density of markers was used in the multi-breed analyses. Pryce et al. [9] suggested and evaluated considering only the genomic regions that are known to be associated with the traits of interest for prediction of GBV. Shulman et al. [32] reported SNP on nine chromosomes to be associated with female fertility traits in Finnish Ayrshire bulls, and that the BTA2 gene also contained a SNP that was significantly associated with non-return rate in cows.

Overall, in this study the use of multi-breed instead of single-breed analyses did not increase the accuracies of GEBV in spite of favourable genetic correlations between breeds, especially for milk production and fat content. Thus, high (higher than 0.6) genetic correlations between breeds were needed in this study to achieve slightly higher precisions. Therefore, for traits with moderately high heritabilities, and using existing genomic relationships between breeds, the genetic correlation between breeds might be an indicator of the expected increase in accuracy of GEBV from the use of a multi-breed reference population. In fact, the genetic correlation provides an indication about the concordance of the effect of the QTL on the trait between breeds (e.g., it might be different, or the QTL might be fixed in one breed and segregating in another) and about the concordance of LD between markers and QTL between breeds.

## Conclusions

A model fitting data on a trait in multiple breeds as correlated pseudo-traits has been presented. The trait that showed the lowest genetic correlation between breeds was female fertility. The use of a multi-breed reference population only increased the accuracy of GEBV for traits and populations that showed the largest correlations between breeds and in the breed with the smallest data set. Accuracies of GEBV for fertility were lower than for other traits and values of the regression of the DYD on the GEBV showed severe underestimation of GEBV for fertility in breed M.

## Competing interests

The authors declare that they have no competing interests.

## Authors' contributions

SK analyzed the data, AL conceived the approach and analyzed the data. SK, MJC, CD, AL interpreted the results and drafted the paper. All authors read and approved the final manuscript.
